# Evaluating the effectiveness of the HPV vaccination programme in England, using regression discontinuity design

**DOI:** 10.23889/ijpds.v9i5.2691

**Published:** 2024-09-18

**Authors:** Jennifer Welsh, Grace Joshy, Rachel Freeman-Robinson, Nicholas Biddle, Emily Banks, Clement Schlegel, Kayla Jordan, Phillip Gould, Paul Kelly, Rosemary Korda

**Affiliations:** 1 Australian National University; 2 Australian Government Department of Health and Aged Care

## Aim

To quantify the impact of the pandemic on fatal burden of disease among those living in residential aged care homes (RACH) in Australia.

## Methods

Using Residential Aged Care data linked to Death Registrations (Jan 2016 to 11 Aug 2022), we estimated years of life lost per person-year (YLL/py) among people living in RACH. Additional linkage of notifications data from the state of Victoria enabled comparisons of average survival times between notified COVID-19 cases and matched non-cases living in RACH using COVID-19.

## Results

COVID-19 deaths were low in 2020 and 2021 (610 and 260 deaths, respectively) but increased in 2022 (3230 deaths). Years of life lost due to COVID-19 was low in 2020 and 2021 (1.2% and 0.5% of the total YLL/py, respectively) but increased substantially in 2022 (8.3% of the total YLL/py). However, there was little evidence that YLLs/py overall increased in the pandemic (range: 0.87 to 0.93 in 2016-2019 and 0.85 to 0.88 in 2020-2022). Results describing differences in survival between notified COVID-19 cases and non-cases are forthcoming. 

## Conclusion

We observed no substantial increase in overall fatal burden of disease among the RACH population during the pandemic period. While COVID-19 case numbers increased in 2022 relative to 2020-2021, this was offset with better survival among cases over time, reflecting, at least in part, decreasing disease severity, high uptake of COVID-19 vaccines and antiviral medications.

